# RRP42, a Subunit of Exosome, Plays an Important Role in Female Gametophytes Development and Mesophyll Cell Morphogenesis in *Arabidopsis*

**DOI:** 10.3389/fpls.2017.00981

**Published:** 2017-06-08

**Authors:** Xiaoyuan Yan, Zongyun Yan, Yuzhen Han

**Affiliations:** State Key Laboratory of Plant Physiology and Biochemistry, College of Biological Sciences, China Agricultural UniversityBeijing, China

**Keywords:** *Arabidopsis*, cell morphogenesis, exosome, female gametophytes, mRNA decay

## Abstract

The exosome complex plays a central and essential role in RNA metabolism. However, current research on functions of exosome subunit in plants is limited. Here, we used an egg cell-specific promoter-controlled CRISPR/Cas9 system to knock out *RRP42* which encodes a core subunit of the *Arabidopsis* exosome and presented evidence that RRP42 is essential for the development of female gametophytes. Next, we designed three different amiRNAs targeting *RRP42*. The *rrp42* knock-down mutants mainly displayed variegated and serrated leaves, especially in cauline leaves. The internal anatomy of cauline leaves displayed irregularly shaped palisade cells and a reduced density of mesophyll cells. Interestingly, we detected highly accumulated mRNAs that encode xyloglucan endotransglucosylase/hydrolases (XTHs) and expansins (EXPAs) during later growth stages in *rrp42* knock-down mutants. The mRNA decay kinetics analysis for *XTH19*, *EXPA10*, and *EXPA11* revealed that RRP42 had a role in the decay of these mRNAs in the cytoplasm. RRP42 is localized to both the nucleus and cytoplasm, and *RRP42* is preferentially expressed in cauline leaves during later growth stages. Altogether, our results demonstrate that RRP42 is essential for the development of female gametophytes and plays an important role in mesophyll cell morphogenesis.

## Introduction

RNA decay is a key step in regulated gene expression. In eukaryotes, the majority of mRNAs undergo decay mainly by a pathway that is initiated by the removal of poly(A)-tail (Couttet et al., 1997; Parker and Song, 2004), and then enters one of two irreversible routes: for one, the 5′ cap is removed by the decapping complex, after which the mRNA body was degraded from the 5′ end by the XRN1 exoribonuclease (Hsu and Stevens, 1993); for another, the unprotected 3′ end is attacked by the 3′→5′ exonucleases (Garneau et al., 2007; Lange et al., 2009; Christie et al., 2011).

The exosome was first identified as a large multisubunit RNase complex that is required for the 3′–5′ processing of ribosomal RNA in yeast (Mitchell et al., 1997), and subsequently in archaea (Koonin et al., 2001; Buttner et al., 2005) and other eukaryotes (Allmang et al., 1999b; Chekanova et al., 2002). The salient feature of the exosome core is the hexameric ring defined by three distinct heterodimers of six RNase PH domain-type proteins: RRP41–RRP45, MTR3–RRP42, and RRP43–RRP46. To form a stable complex, these heterodimers are bridged on one side by three subunits containing S1 and KH domains: RRP40 links RRP45 and RRP46, RRP4 interacts with RRP41 and RRP42, and CSL4 contacts MTR3 and RRP43 (Liu et al., 2006). Although the six PH-ring subunits show clear structural and sequence similarity to RNases, all of these homologs in the human and yeast exosome are inactive because of lacking important catalytic residues (Liu et al., 2006; Dziembowski et al., 2007), and loss of any individual subunit of the nine is lethal, and causes almost identical profiles of RNA-processing defects (Allmang et al., 1999a,b; Liu et al., 2006). In contrast, the exosome subunit RRP41 retained its catalytic competence in the *Arabidopsis* (Chekanova et al., 2000). However, the Rrp44 which is homologous to bacterial RNase II and is responsible for the 3′ exonuclease activity of the exosome (Dziembowski et al., 2007; Barbas et al., 2008; Lorentzen et al., 2008), is stably associated with the core complex in yeast and *Drosophila* but not in human. In *Arabidopsis*, exosome subunits identified by MS/MS revealed that its exosome complex contained three S1 and/or KH domain proteins and six RNase PH domain-containing proteins (Chekanova et al., 2007), but their activities were different from yeast and human. *Arabidopsis* RRP41 is essential for development of the female gametophyte. The *rrp41* female gametophytes arrested after the first mitosis and less frequently at one-nucleate, four-nucleate, or later stages. RRP4 was required for postzygotic development, *rrp4* mutant seeds arrested at early stages of embryogenesis. Loss of CSL4 almost had no effects on the integrity or function of the *Arabidopsis* exosome (Chekanova et al., 2007), and RRP45 is encoded by duplicate genes: *RRP45A* and *RRP45B*. *rrp45a* has no visible defect while *rrp45b* displayed a reduction of cuticular wax loads on the stem and silique. Complete loss of RRP45 function in *Arabidopsis* is lethal (Hooker et al., 2007). In addition, RRP41 homolog RRP41L plays an important role in seed germination and early seedling growth by mediating special mRNA decay in *Arabidopsis* (Yang et al., 2013). RRP44A, the homolog of Rrp44/Dis3, is required for female gametophyte and early embryogenesis (Kumakura et al., 2013). All these data indicate that the subunit of exosome in *Arabidopsis* probably has different functions for plant growth and development (Lange and Gagliardi, 2010). However, the functions of other subunits not discussed above are still unclear in *Arabidopsis*.

Here, we used an egg cell-specific promoter-controlled CRISPR/Cas9 system to knock out *RRP42* and present evidence that RRP42 is essential for the development of female gametophytes in *Arabidopsis*. Next, we obtained three *rrp42* knock-down mutants using artificial microRNA technique: *a42-1*, *2*, *3*. These *rrp42* knock-down mutants mainly displayed variegated and serrated leaves, in which the shape of palisade cell was seriously aberrant. We detected highly accumulated mRNAs that encode xyloglucan endotransglucosylase/hydrolases (XTHs) and expansins (EXPAs) in these mutants. The mRNA decay kinetics analysis further confirmed RRP42 function in the cytoplasm. Altogether, our results demonstrate that RRP42 plays an important role in mesophyll cell morphogenesis and proliferation, especially in cauline leaves.

## Materials and Methods

### Plant Materials and Growth Conditions

In all experiments, *Arabidopsis* ecotype Columbia was used as the wild-type (WT) control. All plant seeds were germinated on MS medium supplemented with agar (1%) and sucrose (3%) at pH 5.8. All plants were grown in soil at 22°C with a 16 h:8 h, light:dark photoperiod.

### Construction of Transforming Vectors

For the *rrp42* null mutant, one sgRNA target (C1: CCAACAGCTGAACCGACATTTGG) in *RRP42* gene was selected and cloned into the pHSN401 (Xing et al., 2014). For the largest possibility of getting the non-mosaic mutants, we cloned target C1 into the pHEE401 vector as described by Wang et al. (2015) later. In addition, we also selected another gRNA target (C2: AGTTCACTTCAACCCGATAAAGG) in *RRP42* gene, and generated another pHEE401 vector with two gRNA expression cassettes targeting the two adjacent sites (C1 and C2) of *RRP42* gene (Wang et al., 2015). The construct was transformed into WT plants by the floral dip method (Clough and Bent, 1998). The putative transformants were screened on MS plates contained with 25 μg ml^-1^ hygromycin B. To detect mutation on targeted sequence, the genomic DNA was isolated from rosette leaves of about 20-day-old T1 transformants. For the sequence analysis of target C1, a 526 bp genomic DNA region containing the target site was amplified by PCR using the primers 42LP (5′-GGCTCTAGGCTAATGGTTCAG-3′) and 42RP (5′-CTGCTCCACTTTTGCCACCCA-3′). We used restriction endonuclease PvuII to digest PCR products for primary screens and obtained a few candidate lines for sequence analysis. For the target C2, we sequenced it directly.

For the *rrp42* knock-down mutants, we designed three different amiRNAs (amiRNA1: TTTCGTTTGGTTAACCCGCAT; amiRNA2: TTTCGTTTGGTTAACCGACAT; amiRNA3: TATATAGATACAGCTGCGCTC) to knockdown the expression levels of *RRP42* in *Arabidopsis* using WMD (web microRNA designer)^1^. Then, according the sequence of amiRNAs designed, we acquired the corresponding primers (I–IV) of amiRNA1, amiRNA2, and amiRNA3 (Supplementary Table S1). The amiRNA foldback fragments were generated using the miR319a vector as a template and the following primers for amplification: A:5′AATTATCTAGAACACACGCTCGGACGCAT-3′. B:5′-AATTAATCCCATGGCGATGCCTT-3′. Each amiRNA corres-ponding primers was designed using the Web MicroRNA Designer 3 oligo design algorithm, and then ligated into pMDC99-32A vectors which harbor a dual 35S promoter. Detailed information regarding the use of overlapping PCR and each primer set is available on the Web MicroRNA Designer 3 web-site. Transformation was performed as described above.

### The Observation of Gametophytes Development

For the observation of ovules, inflorescences were harvested and fixed in 4% glutaraldehyde (in 12.5 mM cacodylate, pH 6.9), and a vacuum was applied for the initial 1 h, after which they were in fixative overnight at room temperature. Then, the inflorescences was dehydrated through a common ethanol series with 30 min per step. After the dehydration, the dehydrated inflorescences was cleared in 1:2 (v/v) benzyl alcohol:benzyl benzoate overnight at room temperature. The dissected pistils were mounted with immersion oil, and observed using a Zeiss LSM710 META confocal laser scanning microscope with a 488 nm argon laser.

For DAPI staining of pollen grains, pollen grains from dehisced anthers were dissected and stained in DAPI solution (PIPES 50 mM, DAPI 5 mg/ml, DMSO 10%, EGTA 5 mM, and NP-40 0.1%) and incubated for 15 min. And then, the stained pollen grains were observed on a Leica HQ stereomicroscope equipped with a 40× optic.

### Morphology and Chlorophyll Content Analysis

The leaf serration was quantified according to Hasson et al. (2011). We took the leaves of the plants and took pictures of them, and then we used the Digimizer software to measure the leaf area.

Chlorophyll was isolated from the leaves and measured according to a previously described method (Grbic and Bleecker, 1995). Extracts were obtained from 100 mg of fresh tissue from the first and second cauline leaves from 42-day-old plants and immerged in 2 mL of 80% (v/v) acetone overnight at room temperature. Chlorophyll was measured by Elisa (Power Wave XS2). Chlorophyll content analysis were repeated at least in three independent experiments.

### Isolation of RNA and qPCR

Total RNA was extracted according to the protocol of Oñate-Sánchez and Vicente-Carbajosa (2008). One microgram of RNA was used as the template to produce cDNA with the TaKaRa oligo(dT) primer and M-MLV reverse transcriptase. We used the SYBR Premix Ex Taq (TaKaRa, Japan) and the ABI 7500 Real Time PCR to perform quantitative real-time PCR (qRT-PCR). *AT4G34270* was used as an internal control. The qRT-PCR analysis used two technical replicates and three biological replicates. The primers used in qRT-PCR are shown in Supplementary Table S2.

### Microscopic and Ultrastructural Analyses

The density of mesophyll cells and chlorophyll fluorescence analysis were observed using a Leica HQ and Zeiss 510 META confocal laser scanning microscope, respectively. Trypan blue staining was performed as described in Koch and Slusarenko (1990). For leaf anatomy light microscopy, the first cauline leaf of 6-week-old WT, *a42-1* and *a42-3* plants were fixed in formaldehyde/glutaraldehyde fixative (1% [v/v] glutaraldehyde and 4% [w/v] paraformaldehyde in 0.05 mol L^-1^ phosphate buffer, pH 7.2). Then, the tissues were dehydrated in ethanol and embedded in Spurr’s resin (SPI-CHEM). Thin sections were cut on a microtome (Leica EM UC7), and observed using light microscopy (ZEISS Scope A1). Materials were also sliced to perform the experiment of ultrathin sections (LKB-8800). Before being examined with a transmission electron microscope (JEM-123O), they were stained with alkaline lead citrate and uranyl acetate. For scanning electron microscopy, plant material was prepared according to a previously described method (Serrano-Cartagena et al., 2000). We used a scanning electron microscope (Hitachi S-3400N) to take micrographs.

### RNA Decay Analysis

RNA decay assays in WT, *a42-1* and *a42-3* mutants were measured in rosette leaves of 6-week-old plants. This protocol was adapted from a previous report from Johnson et al. (2000). Leaves were incubated in buffer (1 mM KCl, 15 mM sucrose, 1 mM sodium citrate, 1 mM Pipes, pH 6.5) for 30 min before addition of cordycepin (150 mg ml^-1^, Sigma). About 30 s vacuum was applied to the all samples. Four leaves were frozen in liquid nitrogen every 15 min for 45 min, and then stored at -80°C for the use of RNA isolation and qRT-PCR.

### Subcellular Localization of RRP42-Fused GFP Protein

To express the RRP42-GFP fusion protein under the control of the 35S promoter, the full-length coding sequence except the stop codon was cloned from the cDNA of WT plants using the primers: GFP-LP (5′-TCTAGAATGGGGCTTTCTCTTGGGGA-3′) and GFP-RP (5′-GGTACCAGATTCGTCTTCGCAGGCCT-3′). The PCR products were fused to the pSuper 1300-GFP vector. The GFP is tagged at its C terminus. The protoplast extraction and plasmid transformation procedures were performed according to a previously described method (Kim and Somers, 2010). We used the floral dip method to obtain the stable *Arabidopsis* transformants, and putative transgenic plants were screened on MS plates containing 25 mg L^-1^ hygromycin. We used a Zeiss 510 META confocal laser scanning microscope to observe the GFP fluorescence of the transgenic plants and the RFP and GFP fluorescence of the transgenic protoplasts. To confirm that the localization of RRP42 was unaffected by GFP fusion, the 35S:RRP42 vector was transformed into the WT plants. The *35S:RRP42* stable *Arabidopsis* transformants displayed no obvious phenotype with the *35S:RRP42:GFP* stable *Arabidopsis* transformants.

### Assay of GUS Activity in Transgenic Plants

For the GUS staining, the *RRP42* promoter:GUS gene was constructed using PCR amplification of a fragment 1533 bp upstream from the initial codon of *RRP42*. We used the following primers for amplification: PRO-LP (5′-CCCAAGCTTATGTGTTTTCATCAGTCCTTACCG-3′) and PRO-RP (5′-GCGTCGACCACTAATACTACACAGAGAACG-3′); the fragment was then cloned into a pCAMBIA 1391 vector. Transformation was performed as described above. Ovules for microscopy were performed as described by Wu et al. (2006). The materials were observed using a microscope (Olympus SZX16-DP72), and a Canon digital camera (PowerShot G12) recorded the digital images.

## Results

### RRP42 Is Essential for the Development of Female Gametophytes

**FIGURE 1:**
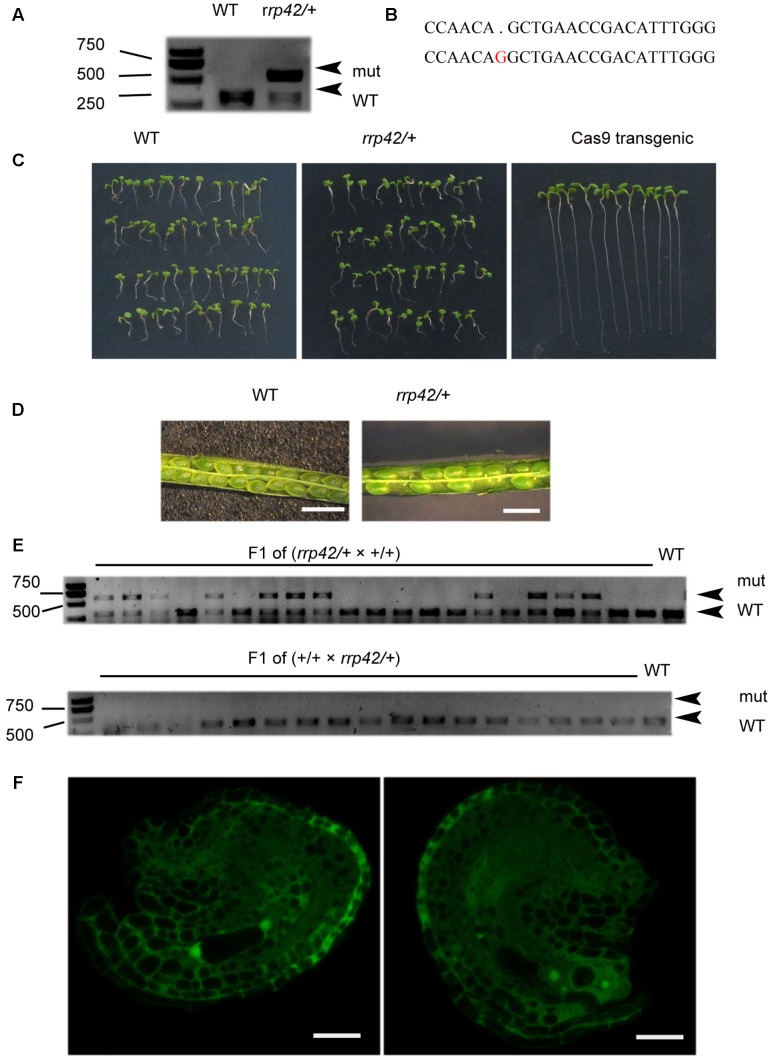
Characterization of the *rrp42/RRP42*. **(A)** PvuII (cleavage sit:CAG/CTG) restriction endonuclease digestion of a RRP42-specific PCR product from WT(+/+) and *rrp42/RRP42* (*rrp42/+*) rosette leaves of 20-day-old plants. **(B)** Sequencing of *At3g07750* in *rrp42/RRP42* (below) and WT (above). **(C)** Separation of the Cas9 gene from the mutant. **(D)** The semi-sterility of *rrp42/RRP42*. Bars = 1 mm. **(E)** PvuII restriction endonuclease digestion of a RRP42-specific PCR product from the F1 progeny of *rrp42/+* × WT (top) and the F1 progeny of WT × *rrp42/+* (bottom). **(F)** *rrp42* female gametophytes at the two-nuclear stage (left) and a mature embryo sac with four-cell (right). Bars = 20 μm.

To investigate the role of exosome component RRP42 (AT3G07750) in *Arabidopsis* development, we used an egg cell-specific promoter-controlled (EPC) CRISPR/Cas9 system to acquire *rrp42* mutant (Wang et al., 2015). The pHEE401-42 construct with one gRNA was transformed into WT plants, only three non-mosaic mutants were obtained of nearly 300 T1 lines. We chose one line (named as *pHEE42-1*) which was identified as heterozygotes for further study. In the heterozygote *rrp42/RRP42*, a G was inserted into the Cas9 editing targets, which were located at 255 and 256 bp in the coding regions of *RRP42*, leading to reading frame shifts (**Figures 1A,B**). To eliminate the influence of the Cas9 gene, we isolated *rrp42/RRP42* on MS plates containing hygromycin (**Figure 1C**). The non-resistant plants were used for further study. RRP42 was essential for development of the female gametophyte. The selfed heterozygote *rrp42/RRP42* produced seeds and aborted ovules in a 1:1 (252:265) ratio (**Figure 1D**; three non-mosaic mutants showed identical phenotypes), and in the progeny of selfed heterozygote *rrp42/RRP42*, the proportion of heterozygous and WT plants was almost 1:1 (50:47). We also found that the *rrp42* mutant allele was normally transmitted through the male parent, but couldn’t transmitted through the female (**Figure 1E**). Furthermore, according to the previous description of Smyth et al. (1990), most of embryo sacs were at the four-celled stage at flower developmental stage 14 in WT pistils. While the *rrp42/RRP42* female gametophytes arrested (*n* = 134) after the first mitosis (two-nucleate stage, 46.3%; **Figure 1F**) and less frequently at one-nucleate (2.2%), four-nucleate (8.9%). Additionally, there was no obvious morphological difference in *rrp42/RRP42* and WT pollen grains (Supplementary Figure S1A). These observations suggest that the synchrony of female gametophyte development was impaired in *rrp42* pistils.

We also transformed the construct pHSN42 with one gRNA and pHEE-42 with two gRNA into WT plants, respectively. The heterozygote *rrp42/RRP42* from pHSN42 construct transformation was named as *pHSN42-1* while another heterozygote from the transformation of pHEE-42 construct with two gRNA was named as *pHEE-2g-42-1*. They showed the identical phenotype as that in *pHEE42-1* heterozygote. In these three heterozygotes *rrp42/RRP42*: *pHEE-42-1*, *pHSN42-1*, and *pHEE-2g-42-1*, the mutational pattern were different with each other (**Figure 1B** and Supplementary Figures S1B,C). These data indicated that the null mutation of *rrp42* is lethal and RRP42 has an essential role for development of the female gametophyte in *Arabidopsis*.

### Generation of Three *rrp42* Knock-Down Mutants

**FIGURE 2:**
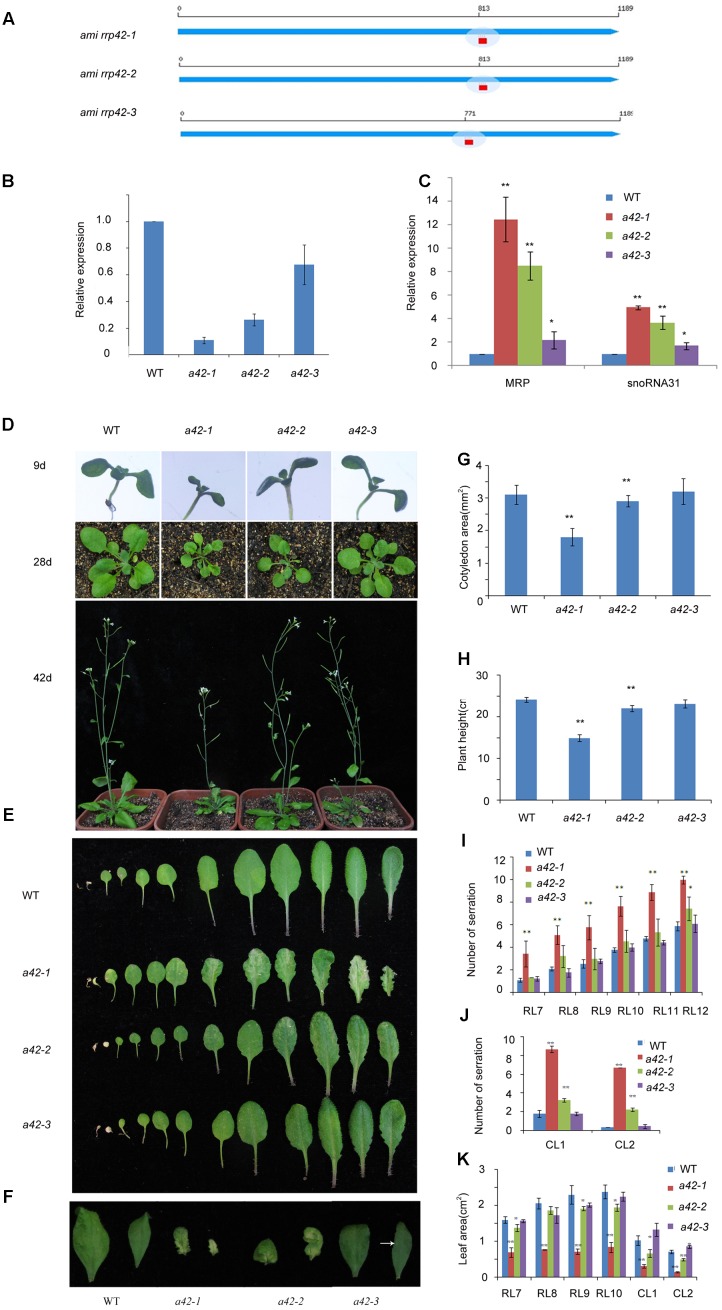
Phenotypic characterization of WT, *a42-1*, *a42-2*, and *a42-3* plants. **(A)** The position of three target sequences of *RRP42*. **(B)** Expression of *RRP42* in WT, *a42-1*, *a42-2*, and *a42-3* as analyzed by qRT-PCR. **(C)** Expression of MRP RNA and snoRNA31 in WT, *a42-1*, *a42-2*, and *a42-3* as analyzed by qRT-PCR. **(D)** Morphological traits of 9-, 28-, and 42-day-old seedlings. **(E)** Morphological traits of 42-day-old rosette leaves. **(F)** The first and second cauline leaves of 42-day-old WT, *a42-1*, *a42-2*, and *a42-3* plants. **(G)** Cotyledon area of 9-day-old seedlings. **(H)** The height of 6-week-old plants. **(I)** Analysis of serrations of rosette leaves (RL) 7 to 12. **(J)** Analysis of serrations of cauline leaves (CL) 1 and 2. **(K)** Analysis of the area of rosette leaves (RL) 7 to 10 and cauline leaves (CL) 1 and 2. The data are expressed as means ± SD of three biological replicates. At least 30 seedlings per genotype were measured in each replicate. ^∗^*P* < 0.05 and ^∗∗^*P* < 0.01 (Student’s *t*-test) indicate significant differences between mutants and WT plants.

To address the functions of RRP42 during vegetative growth, we designed three different amiRNAs targeting *RRP42* using web microRNA designer^2^ (**Figure 2A**). We obtained at least 50 T1 lines with similar phenotypes. Next, we chose one line from the lines targeted by amiRNA1, amiRNA2 and amiRNA3, respectively, and referred to as *a42-1*, *a42-2*, and *a42-3* for the next step of analysis. Knock-down of *RRP42* expression induced various morphological abnormalities, which were specifically evident on both the cauline and rosette leaves (**Figures 2D–F**). The abnormal phenotypes of *rrp42* knock-down mutants could be grouped into three classes according to the severity of the abnormalities. The levels of *RRP42* in *a42-1* with a severe defect decreased to about 10% of the WT plant, while levels in *a42-3* with a mild defect were about 70% of WT (**Figure 2B**). Thus, the expression levels of *RRP42* were consistent with the severity of *rrp42* knock-down mutant defects. These results indicated that the defects of *rrp42* knock-down mutants were induced by the repressive activity of RRP42.

We also tested the expression levels of *MRP* and *snoRNA31*, which were the known nucleus target RNAs of exosome and accumulated in *rrp4^iRNAi^*, *rrp41^iRNAi^*, and *rrp44a* mutant (Chekanova et al., 2007; Kumakura et al., 2013). The levels of *MRP* and *snoRNA31* were significantly increased in different degrees in three mutants (**Figure 2C**). The most accumulation of both RNAs was in *a42-1*, while the least accumulation was in *a42-3*. These data further show that the phenotype is the result of RRP42 downregulation.

### The *rrp42* Knock-Down Mutants Displayed Primarily Variegated and Serrated Leaves during Later Growth Stages

Leaf morphologies of *rrp42* knock-down mutant are shown in **Figure 2**. The cotyledons of *rrp42* knock-down seedlings displayed wrinkled surface and a reduced leaf size (**Figures 2D,G**), and the root growth was no obviously affected in mutant seedlings (Supplementary Figure S2A). The most observable defects in *rrp42* knock-down mutant were the variegated and serrated cauline leaves of older plants (**Figures 2F,J**). These defects were also visible in rosette leaves (**Figures 2E,I**). The *a42-1* mutant with a severe defect was serrated and distorted beginning at the seventh or eighth rosette leaf, leaf size in *a42-1* mutant was also reduced (**Figures 2I,K**). The *a42-2* mutant with a moderate defect was beginning at the eleventh or twelfth rosette leaf (**Figure 2I**), while the *a42-3* with a mild defect was almost normal except for one or two weakly variegated cauline leaves (**Figure 2F**). The abnormal phenotypes of *rrp42* knock-down mutant become more severe at later growth stages. After bolting, the stem of *a42-1* was a little twisted compared with WT, and the final height of *a42-1* was below that of the WT plant (**Figure 2H**). These results suggested that knock-down of *RRP42* seriously affected the plant growth and leaf development.

**FIGURE 3:**
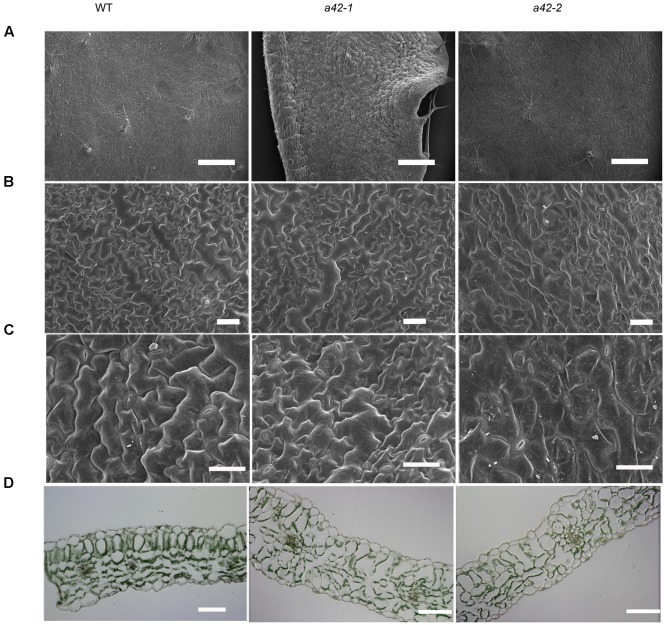
Scanning electron micrographs and anatomical structure of CL1 (the first cauline leaf) in WT, *a42-1*, and *a42-2* leaves. **(A)** The section of CL1 adaxial epidermises. Bars = 4 mm. **(B)** Detail of CL1 adaxial epidermises. Bars = 0.4 mm. **(C)** Detail of CL1 abaxial epidermises. Bars = 0.3 mm. **(D)** Semithin section of CL1. Bars = 50 μm. Leaves were collected from 6-week-old plants.

**FIGURE 4:**
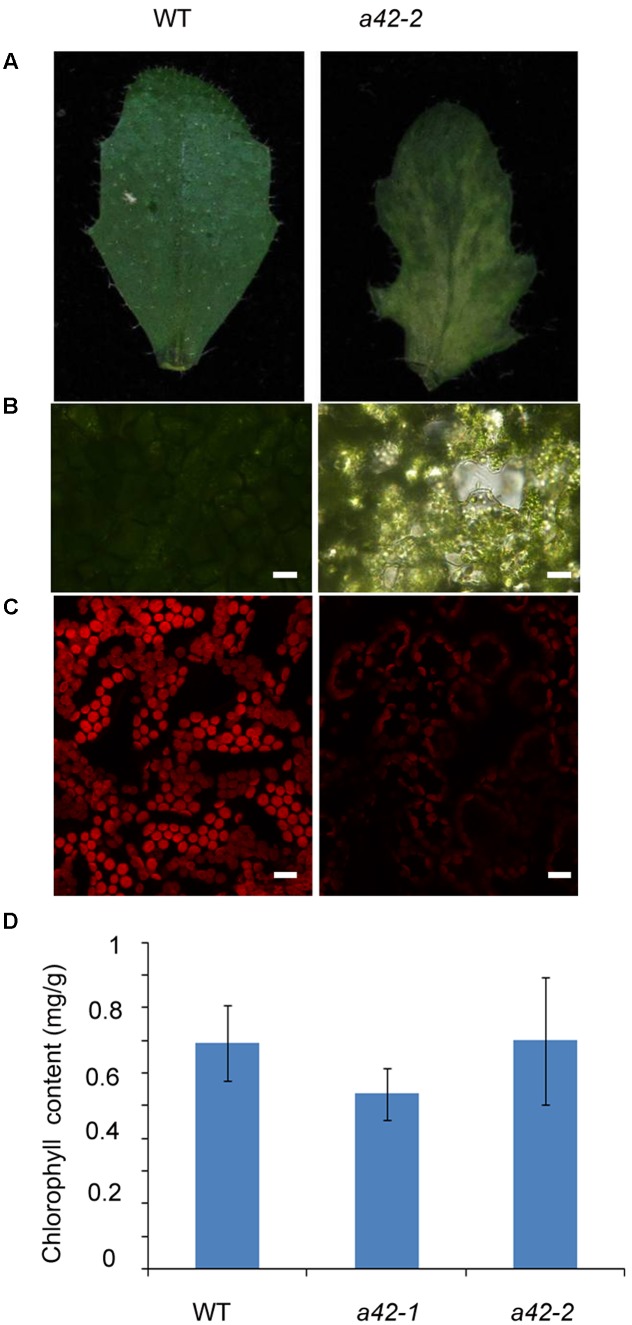
Reduced cell density in the mesophyll of *a42-2* cauline leaves. **(A)** Six-week-old cauline leaf 1 of WT and *a42-2* plant. **(B)** Microscopy analysis of mesophyll cells of the first cauline leaf. Bars = 20 μm. **(C)** Mesophyll cells chlorophyll fluorescence of the first cauline leaf. Bars = 15 μm. **(D)** Chlorophyll content of the first and second cauline leaves of WT, *a42-1*, and *a42-2* plants. The data are expressed as means ± SD of three replicates. At least 30 seedlings per genotype were measured in each replicate.

Scanning electron microscopy showed that the surface of the mutant leaves was wrinkled, extremely so in the case of the *a42-1*, whose lamina was completely crumpled (**Figure 3A**). Nevertheless, no obvious differences compared to the WT were observed for the size and morphology of the *a42-1* adaxial and abaxial epidermal cells (**Figures 3B,C**). We also analyzed internal leaf anatomy by means of cross sections, *a42-1* and *a42-2* leaves displayed disruptions in cell arrangement and morphology, as well as the overall leaf shape. There were larger air spaces between cells from mutants compared to the WT and the area of palisade cell wall surface was larger than that in WT leaves because of the contraction in the middle position of mutant cells (**Figure 3D**). According to the microscopic and chlorophyll fluorescence analysis of mesophyll cells of the first and second cauline leaves, we found a reduced density of mesophyll cells in *a42-2* (**Figures 4A–C**). However, we found no significant reduction of chlorophyll content in the first and second cauline leaves from 6-week-old *a42-1* or *a42-2* mutant plants as compared with WT plants (**Figure 4D**). We did not observe increased cell death in *rrp42* knock-down mutants by trypan blue staining (Supplementary Figure S3), therefore, we concluded that the increased air spaces and irregular cell morphologies were responsible for the variegation and the distorted surface observed in *rrp42* knock-down mutant leaves.

We also examined leaf chloroplast ultrastructure in the first variegated cauline leaf of *a42-1* and WT by transmission electron microscopy and found that chloroplasts in *a42-1* exhibited reduced starch grains, but they were similar in numbers, size, and morphology to those of WT (Supplementary Figures S4A–D). Only a few chloroplasts in the *a42-1* mutant displayed enlarged thylakoid lamellas (Supplementary Figure S4E), a trait never observed in WT.

We also generated a construct harboring *AT3G07750* the full-length coding sequence under the control of 35S promoter and the GFP was tagged at its C terminus. At least ten homozygous transgenic over-expression (OE) lines were obtained, and *RRP42* transcript levels were measured using qRT-PCR (Supplementary Figure S2B). Under normal conditions, the morphologies of OE1 and OE2 displayed no obvious difference with WT plants.

### *RRP42* Is preferentially Expressed in Leaves, and Localized to Both the Cytoplasm and Nucleus

**FIGURE 5:**
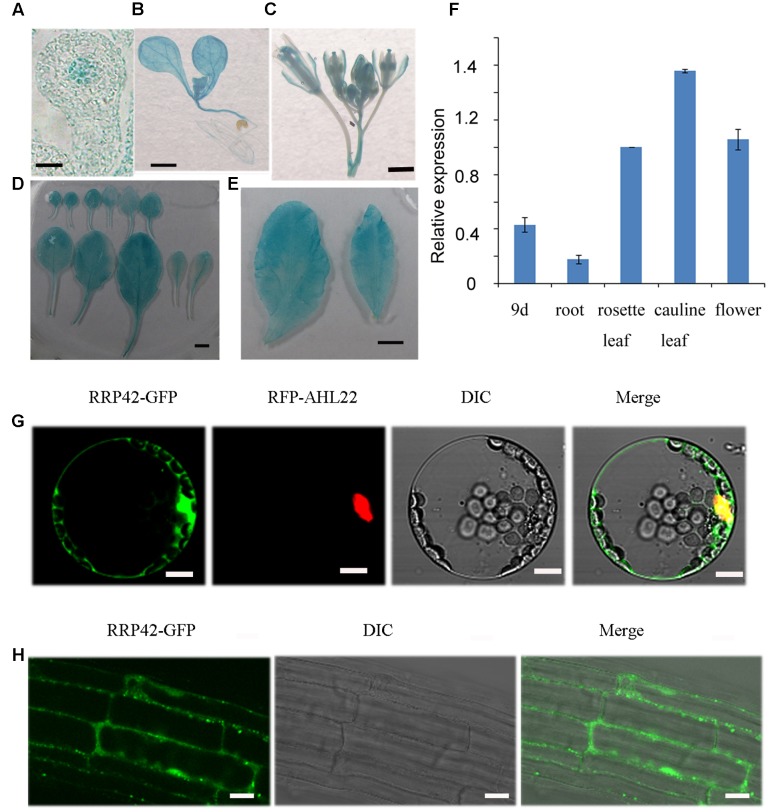
Expression pattern of RRP42 and subcellular localization of RRP42-GFP protein. **(A)** The ovule of stage 3-I. Bar = 5 μm. **(B)** Nine-day-old seedlings. Bar = 1 mm. **(C)** Six-week-old flowers. Bar = 1 mm. **(D)** Six-week-old rosette leaves 1–11. Bar = 5 mm. **(E)** Six-week-old cauline leaf 1 (left) and 2 (right). Bar = 5 mm. **(F)** Expression of *RRP42* in various organs determined by qRT-PCR. Total RNA was isolated from roots, 7 to 8 rosette leaves, cauline leaves and flowers of 6-week-old WT plants and 9-day-old WT seedlings. Transcript levels were quantified by qRT-PCR against *AT4G34270*. The data are expressed as means ± SD of three independent biological determinations. **(G)** Intracellular distribution of RRP42-GFP and RFP-AHL22 proteins in living *Arabidopsis* protoplasts. Bars = 10 μm. **(H)** Intracellular distribution of RRP42-GFP proteins in root cells of 7-day-old stable *Arabidopsis* transformants. Bars = 20 μm.

To investigate the spatial and temporal expression patterns of *RRP42* in plant tissue, a vector in which a 1533-bp promoter fragment of *RRP42* was fused with the GUS gene was constructed. Seven independent transgenic lines were obtained, and at least four lines were detected. GUS activity was observed in all of the organs. We found that *RRP42* was strongly expressed in the leaves (**Figures 5B–F**). It also had a high expression in the ovules (**Figure 5A**). The qPCR analysis also showed that *RRP42* were expressed at higher levels in the cauline leaf (**Figure 5F**). A similar expression pattern was observed in four independent lines. These data were consistent with the results that the RRP42 may play an important role in female gametophytes and leaf development in *Arabidopsis*.

To investigate the intracellular localization of RRP42, we constructed an RRP42-GFP fusion protein expressed under the control of the cauliflower mosaic virus 35S promoter. RRP42-GFP and red fluorescent protein (RFP)-AHL22, an AT -rich DNA sequence (AT)-hook motif nucleus-localized protein (Xiao et al., 2009), were transiently coexpressed in living *Arabidopsis* protoplasts. In contrast to the nuclear distribution of RFP-AHL22, we found that the RRP42-GFP fusion protein was localized to both the cytoplasm and nucleus (**Figure 5G**). Consistent with this, the localization of RRP42-GFP was also in the cytoplasm and nucleus in the 9-day-old stable *Arabidopsis* transformants root cells (**Figure 5H**).

### RRP42 Functions in Cytoplasmic mRNA Decay

**FIGURE 6:**
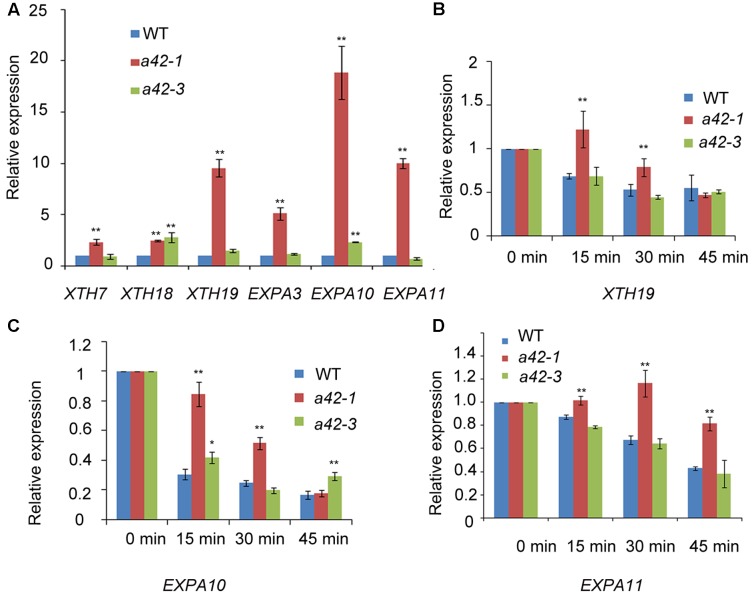
qRT-PCR analysis of transcripts of xyloglucan endotransglucosylase/hydrolases, and expansins as well as RNA decay comparison of *XTH19*, *EXPA10*, and *EXPA11* in WT, *a42-1*, and *a42-3*. **(A)** Transcription levels of XTHs and expansins. **(B–D)** Relative expression is shown for *XTH19*, *EXPA10*, and *EXPA11* in WT, *a42-1*, and *a42-3* before the addition of cordycepin at 0 min and 15, 30, and 45 min after the addition of cordycepin. Rosette leaves 7 to 12 of 6-week-old plants were used for treatment and then for RNA extraction. Transcript levels were quantified by qRT-PCR against *AT4G34270*. The data are expressed as means ± SD of three independent biological determinations. ^∗^*P* < 0.05 and ^∗∗^*P* < 0.01 (Student’s *t*-test) indicate significant differences between mutant and WT plants.

The phenotypic defects of *rrp42* knock-down mutants prompted us to use qRT-PCR to measure the level of transcripts that encode proteins participating in photosynthesis, starch synthesis, cell wall assembly, and leaf morphogenesis. The 42 days rosette leaves of WT, *a42-1* and *a42-3* were used to perform the analysis. We found that expression of the XTHs and EXPAs, both involved in cell wall assembly, were at least two-fold higher in *a42-1*. The expression of these mRNAs were almost unchanged or a little higher in *a42-3* with a mild phenotype (**Figure 6A**). Most noticeably of all, the expression of *XTH19*, *EXPA10*, and *EXPA11* were up to about 20-fold in *a42-1* compared with WT (**Figure 6A**). Additionally, the expression of the genes related to photosynthesis and starch synthesis were almost normal in the two mutants as compared with WT (**Table 1**). While the expression of *CUC1*, a gene involved in leaf blade margin, was increased about six-fold in *a42-1* (**Table 1**). The expression levels of *CUC2* and *CUC3* were nearly unchanged among these mutants.

**Table 1:** The levels of transcripts that encode proteins related to photosynthesis, starch synthesis, and leaf morphogenesis in WT, *a42-1* and *a42-3* plants.

AGI	Symbol	WT	*a42-1*	*a42-3*
**Transcripts encoding proteins related to photosynthesis andstarch synthesis**				
ATCG00490	RBCL	1	0.68 ± 0.14	1.37 ± 0.31
ATCG00020	PSBA	1	0.42 ± 0.04	0.68 ± 0.24
ATCG00120	ATPA	1	1.11 ± 0.06	0.87 ± 0.04
ATMG00070	NAD9	1	0.68 ± 0.08	0.91 ± 0.06
AT5G46110	TPT	1	0.86 ± 0.03	0.68 ± 0.10
AT5G48300	ADG1	1	1.79 ± 0.16	1.62 ± 0.01
AT5G19220	ADG2	1	0.69 ± 0.02	0.95 ± 0.11
**Transcripts containing the NAC domain**				
AT3G15170	CUC1	1	6.29 ± 1.15	4.49 ± 0.51
AT5G53950	CUC2	1	1.28 ± 0.07	1.21 ± 0.03
AT1G76420	CUC3	1	1.48 ± 0.08	1.23 ± 0.02

*qRT-PCR analysis of transcripts (shown in fold change) in 6-week-old WT, *a42-1* and *a42-3* rosette leaves 7 to 12 is shown. The expression level in the WT was set at 1. The means of three replicates of qRT-PCR and SD values are shown. Similar results were obtained when qRT-PCR was performed using a second set of samples*.

To investigate the putative function of RRP42 in the degredation of XTH and EXPA mRNAs, three independent experiments were performed in which excised rosette leaves from *a42-1*, *a42-3*, and WT plants were incubated with cordycepin, a compound that strongly blocked the process of transcription (Zhang et al., 2010). We tested *XTH19*, *EXPA10* and *EXPA11* which were high accumulated in mutant plants. The decay of *XTH19*, *EXPA10*, and *EXPA11* mRNAs in *a42-1* leaves was clearly slower than in WT leaves. As expected, the differences in decay kinetics are evident at the 15 and 30 min point (**Figures 6B–D**), suggesting that RRP42 has a role in the decay of these mRNAs. We also tested the mRNA decay of *EXPL1* (*expansion-like 1*) which has a comparable half life as control (Xu et al., 2006; Xu and Chua, 2009). There were no obviously differences at each point in mutants and WT plants (Supplementary Figure S5). The decay of *XTH19*, *EXPA10*, and *EXPA11* mRNAs was not completely arrested in the two mutants; this could be the consequence of the residual function of RRP42 in the *rrp42* knock-down mutant. Alternatively, RRP42 may not be the only pathway for the decay of these genes at the developmental state tested. In *a42-3* mutant, the rate of these mRNA decay was nearly same or only a little slower compared with WT plant (**Figures 6B–D**), this is consistent with that morphology of *a42-3* rosette leaves was almost normal.

Additionally, to investigate whether the knock-down of *RRP42* could affect the expression levels of mRNAs up-regulated in other exosome subunit mutants, we randomly chose a number of mRNAs up-regulated in the *rrp41* and *rrp4* mutants, and examined their transcript levels in *a42-1* and *a42-3* by qRT-PCR. The results showed that these mRNA levels were almost equal to WT plants. We also examined the expression levels of mRNAs up-regulated in *rrp41l*. As expected, only slight increases or decreases occurred in these mRNA levels compared with WT plants (Supplementary Table S3). Conversely, we also tested the expression of *XTH7*, *XTH18*, *XTH19*, *EXPA3*, *EXPA10*, and *EXPA11* in *rrp41l*. We found that their expression level were basically the same as that of the WT (Supplementary Table S4).

## Discussion

In this study, we used egg cell-specific promoter-controlled CRISPR/Cas9 systems demonstrate that RRP42 is essential for the development of female gametophytes and a homozygous *rrp42* mutant is lethal (**Figure 1**). Furthermore, we acquired several *rrp42* knock-down mutants (**Figure 2**). The cotyledons of *rrp42* knock-down seedlings displayed a wrinkled surface and accumulated anthocyanins in the base of petiole (**Figure 2D**). During later development stages, the cauline and rosette leaves displayed a variegated and serrated phenotype (**Figures 2E,F,I,J**). Consistent with these observations, an expression profile analysis revealed that *RRP42* was preferentially expressed in ovules and cauline leaves (**Figures 5A,F**). The defects in leaf morphogenesis we observed in *rrp42* knock-down mutant (increased air spaces, deformed cells, and reduced numbers of palisade cells) have also been reported in the variegation mutants *msl2-1; msl3-1* (Haswell and Meyerowitz, 2006). Therefore, we concluded that the increased air spaces and deformed cells evident are responsible for the variegation and wrinkled epidermis observed in *rrp42* knock-down mutant leaves. Taken together, all these data indicate that RRP42 plays an important role in the female gametophytes and leaf development.

We also found that the transcripts level that encoded proteins related to cell wall assembly and leaf morphogenesis were highly expressed during the later growth stages of *Arabidopsis* (**Figure 6A** and **Table 1**). XTHs are a family of enzymes that catalyze the hydrolysis and/or molecular grafting of xyloglucans (Nishitani and Tominaga, 1992; Okazawa et al., 1993; Rose et al., 2002; Maris et al., 2009; Han et al., 2016). They play a very important role in the restructuring and construction of load-bearing cross links among cellulose microfibrils and the framework of the cell wall. EXPAs are extracellular matrix proteins that have long been participated in the control of plant growth processes through their functions in modulating cell wall extensibility (Cosgrove, 2005). As described previously, *XTH18* is expressed in differentiating and elongating regions. *XTH19* is expressed in the apical dividing and elongating regions as well as in the differentiation region (Yokoyama and Nishitani, 2001; Vissenberg et al., 2005). *EXPA10*, *EXPA11* are involved in the leaf growth (Cho and Cosgrove, 2000; Li et al., 2003; Goh et al., 2012). The accumulation of these mRNAs, to some extent, may explain the abnormal palisade cell shape and mesophyll cell proliferation in the *rrp42* knock-down mutant. It has been showed that overexpression of *CsExp1* results in disordered arrays and shapes of palisade cells (Pien et al., 2001), which were similar to changes observed in *rrp42* knock-down mutant (**Figure 3D**). *XTH19* and *XTH20* have been shown to promote cell proliferation in pith tissue of incised *Arabidopsis* stems (Pitaksaringkarn et al., 2014).*CUC1*, *CUC2*, and *CUC3* are members of the *NAC* genes and are involved in leaf dissection in the leaf blade margin (Hasson et al., 2011). In the serrated *rrp42* knock-down mutants, the transcript levels of *CUC2* and *CUC3* were almost unchanged, while that of *CUC1* transcripts were increased six-fold compared to WT plants. The phenotype of serration may be a direct or indirect effect of the accumulation of *CUC1* expression.

The 3′–5′ turnover of mRNAs is carried out by the exosome with other factors and RNA helicase (Houseley et al., 2006). In this study, we provide evidence that RRP42 functions in cytoplasmic mRNA decay. We examined transcription levels of *XTH19*, *EXPA10*, and *EXPA11* in 6-week-old rosette leaves treated with cordycepin (**Figures 6B–D**). In WT plants, these mRNA levels decreased rapidly after the addition of cordycepin. However, in the *a42-1* mutant, these mRNA levels decreased much slowly under the same conditions, and the decay of these mRNA was not completely arrested in the *a42-1* mutant. These data suggest that either the *a42-1* mutant retained residual RRP42 function, or that *XTH19*, *EXPA10*, and *EXPA11* mRNA decay were also regulated by other pathways. Taken together, all the above results indicate that RRP42 has functions in cytoplasmic mRNA decay. Thus, we concluded that RRP42 plays an important role in palisade cell morphogenesis and mesophyll cell proliferation, at least partially by mediating some cytoplasmic mRNAs decay in the later growth stages of *Arabidopsis*. The RRP42-GFP fusion experiment indicated that RRP42 was localized to the nucleus and cytoplasm (**Figures 5G,H**), which supports the conclusion that RRP42 functions in the cytoplasm.

Previous work has demonstrated that loss of individual subunits of the exosome leads to different defects in *Arabidopsis*. For example, the *csl4* mutant showed no obvious phenotype, while *rrp4* mutant seeds arrest at an early stage of embryo development. RRP41 plays an important role in development of gametophytes (Chekanova et al., 2007). The *rrp45b* mutant exhibits a reduction of cuticular wax loads on the surface of stem and silique and a complete loss of RRP45 function in *Arabidopsis* is gametophytic lethal (Hooker et al., 2007). The *rrp41l* mutant showed delayed germination and various developmental defects in early development (Yang et al., 2013). In our study, we find that RRP42 is essential for the development of female gametophytes and plays an important role in mesophyll cell morphogenesis. We also detected some mRNAs that were up-regulated in other subunit mutants. There were no notable increase in expression levels of those mRNAs in *a42-1* and *a42-3* compared with WT plants (Supplementary Table S3). And the expression levels of mRNAs up-regulated in *a42-1* were also unchanged in *rrp41l* mutant (Supplementary Table S4). Thus, it seems that each subunit of exosome has partially independent functions in cytoplasm. This hypothesis needs further experimental evidence.

## Author Contributions

YH conceived the research, supervised the experiment. XY designed and performed experiments, and prepared the figures; ZY provided technical assistance; XY and YH wrote the manuscript.

## Conflict of Interest Statement

The authors declare that the research was conducted in the absence of any commercial or financial relationships that could be construed as a potential conflict of interest.

## Footnotes

1 http://wmd3.weigelworld.org

2 http://wmd3.weigelworld.org
