# Supplementary material for: RRP42, a Subunit of Exosome, Plays an Important Role in Female Gametophytes Development and Mesophyll Cell Morphogenesis in Arabidopsis
Source: Front Plant Sci. 2017 Jun 8;8:981. doi: 10.3389/fpls.2017.00981 (PMC5463273; doi:10.3389/fpls.2017.00981)
Supplement: Supplementary file 1 [file Data_Sheet_1.docx]

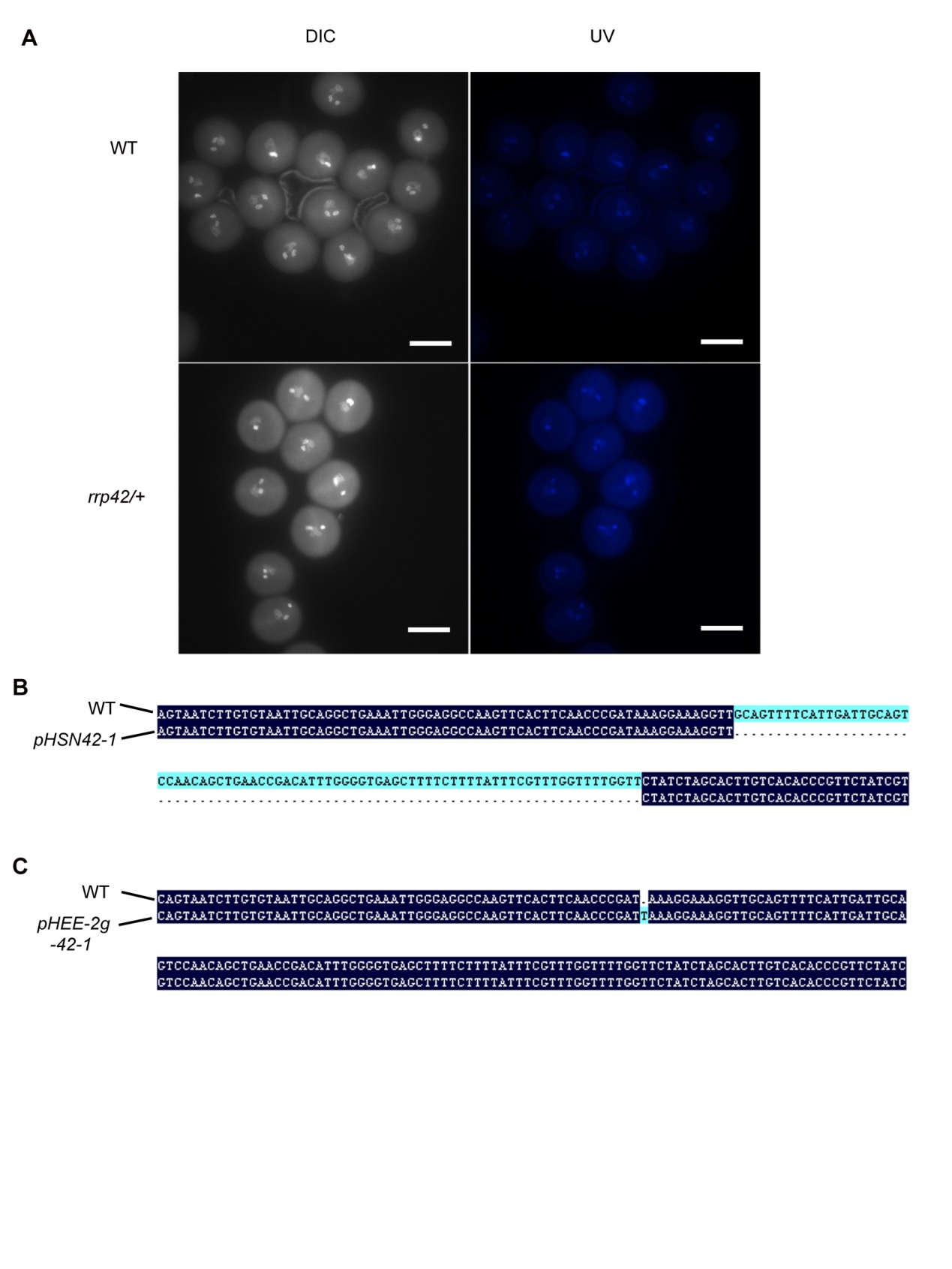


**Supplementary Figure S1. Characterization of the *rrp42/RRP42* heterozegote (A)** Mature pollen stained with DAPI and observed under UV fluorescence. Bars=20μm. **(B)** Sequencing of *At3g07750* in *pHSN42-1* heterozegote. 79 bases were deleted from the 409bp to 487bp in the *At3g07750* DNA. **(C)** Sequencing of *At3g07750* in *pHEE-2g-42-1* heterozegote. A T were inserted into the coding regions of *RRP42* which was located at 216 bp and 217 bp.


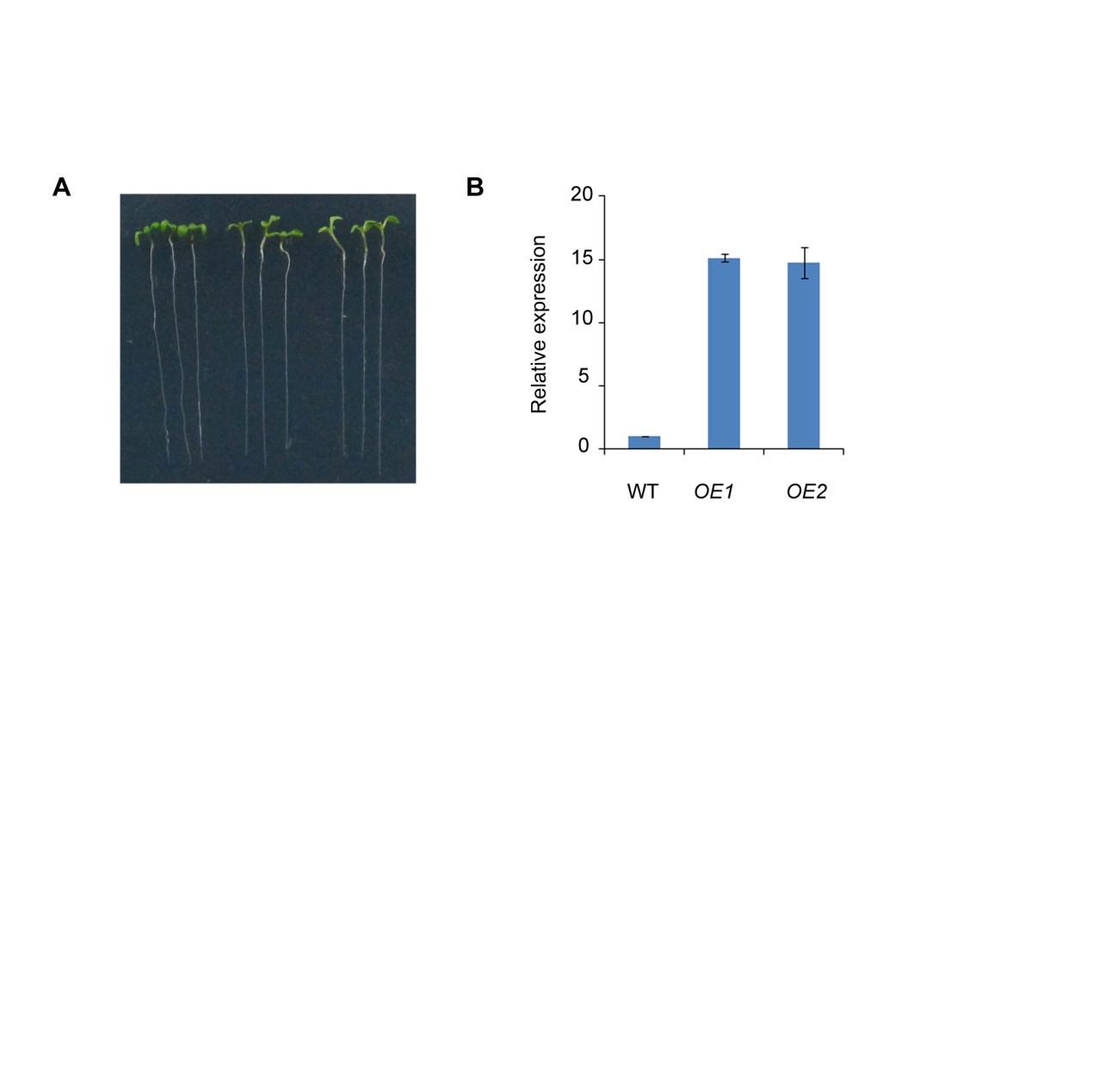


**Supplementary Figure S2. Characterization of *rrp42* knock-down mutants and the overexpression lines. (A)** 9-d-old WT (left), *a42-1* (middle) and *a42-2* (right) seedlings. **(B)** qRT-PCR analysis transcript of *RRP42* in the 9-d-old WT and *OE1*,*OE2* lines. Transcript levels were quantified by qRT-PCR against *AT4G34270*. The data are expressed as means ± SD of three independent biological determinations.


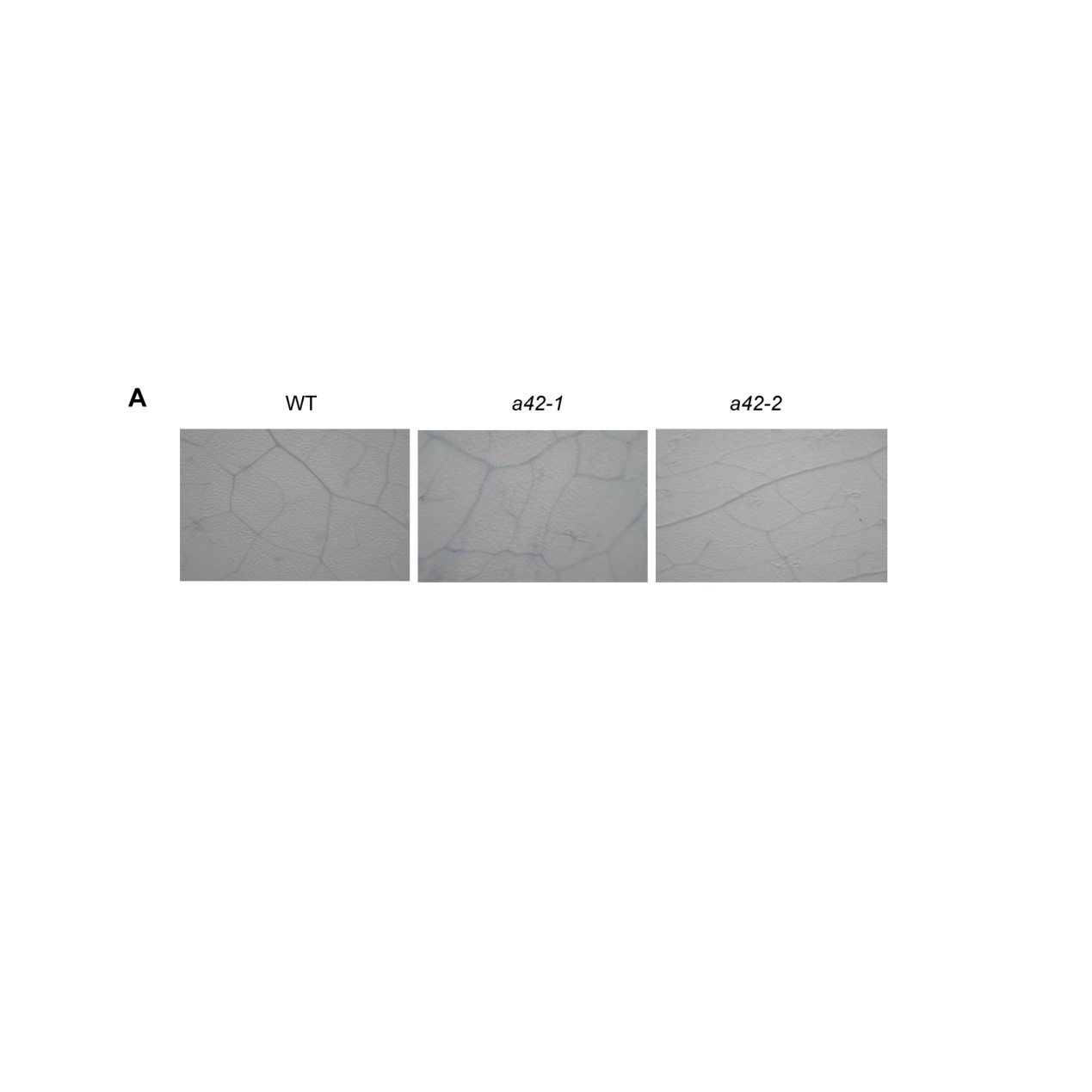


**Supplementary Figure S3. The first cauline leaves of 6-week-old WT, *a42-1* and *a42-2* plants were stained with trypan blue.**


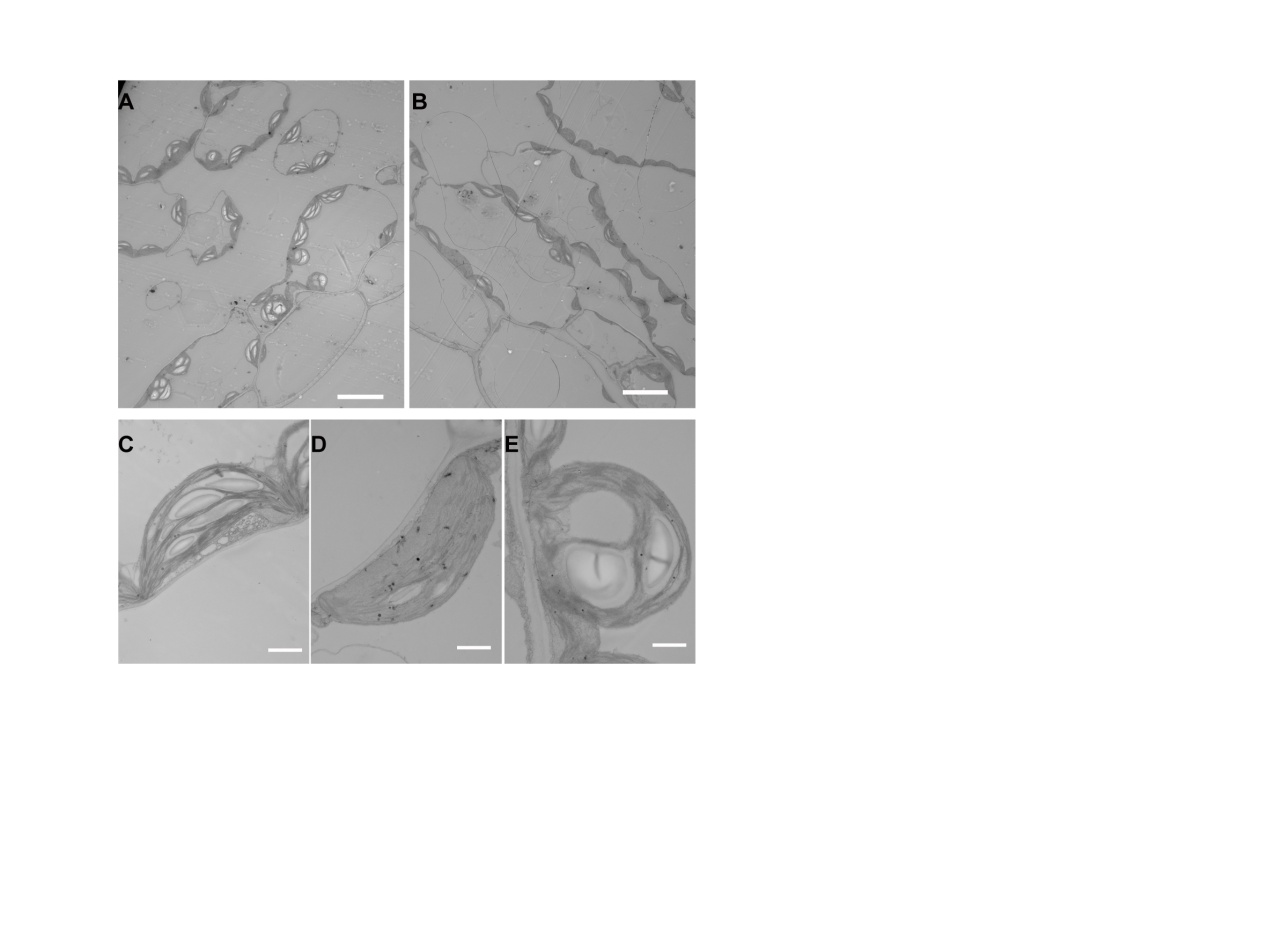


**Supplementary Figure S4. Transmission electron micrographs of WT and *a42-1* chloroplasts.** **(A, C)** Chloroplasts of WT cauline leaf 1. **(B, D)** Chloroplasts of *a42-1* cauline leaf 1. **(E)** One of the chloroplasts occasionally seen in *a42-1* mesophyll cells, showing enlarged thylakoid lamellas. All leaves were collected from 6-week-old plants. (A) and (B) Bars=10μm. (C) to (E) Bars=1μm.


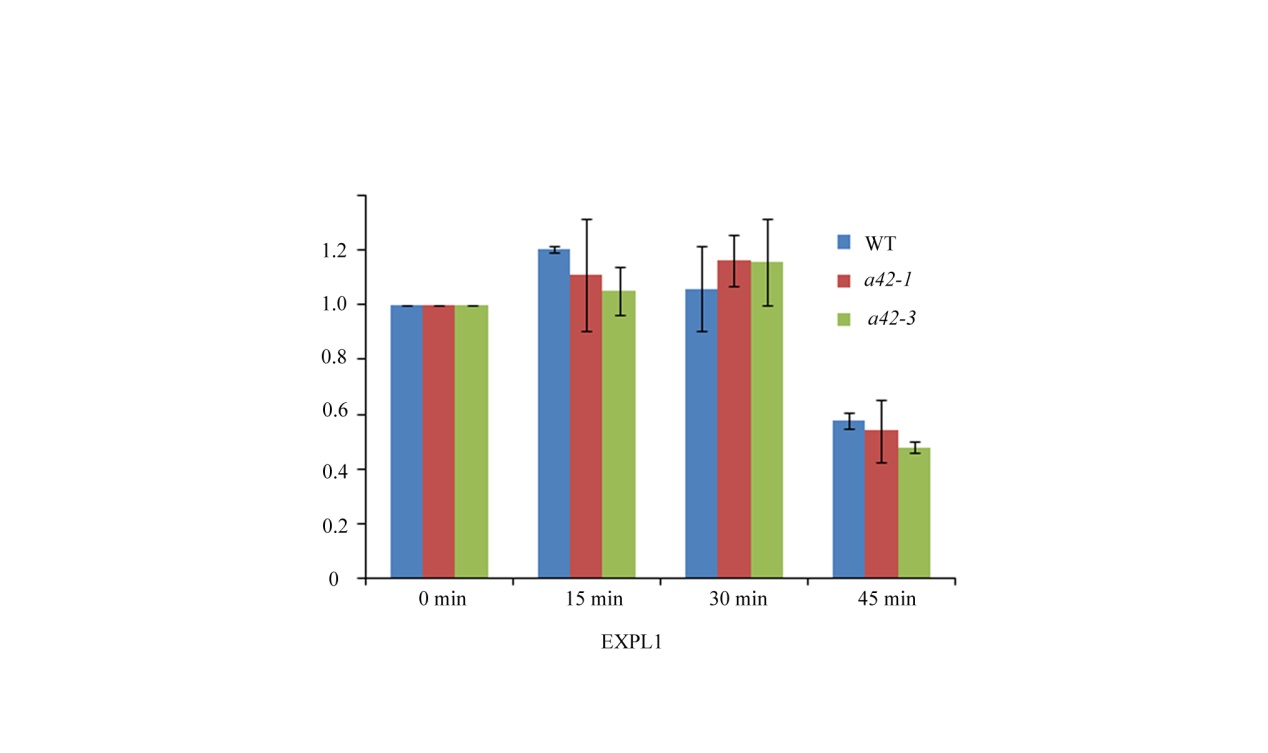


**Supplementary Figure S5. RNA decay comparison of *EXPL1* in WT, *a42-1* and *a42-3*.**

**Supplementary Table S1**

| Primers were designed using the Web MicroRNA Designer 3 oligo design algorithm (5 '-3 ') | | |
| --- | --- | --- |
| amiRRP42-1 | I miR-s | gaTTTCGTTTGGTTAACCCGCATtctctcttttgtattcc |
|  | II miR-a | gaATGCGGGTTAACCAAACGAAAtcaaagagaatcaatga |
|  | IIImiR*s | gaATACGGGTTAACCTAACGAATtcacaggtcgtgatatg |
|  | IVmiR*a | gaATTCGTTAGGTTAACCCGTATtctacatatatattcct |
| amiRRP42-2 | I miR-s | gaTTTCGTTTGGTTAACCGACATtctctcttttgtattcc |
|  | II miR-a | gaATGTCGGTTAACCAAACGAAAtcaaagagaatcaatga |
|  | IIImiR*s | gaATATCGGTTAACCTAACGAATtcacaggtcgtgatatg |
|  | IVmiR*a | gaATTCGTTAGGTTAACCGATATtctacatatatattcct |
| amiRRP42-3 | I miR-s | gaTATATAGATACAGCTGCGCTCtctctcttttgtattcc |
|  | II miR-a | gaGAGCGCAGCTGTATCTATATAtcaaagagaatcaatga |
|  | IIImiR*s | gaGAACGCAGCTGTAACTATATTtcacaggtcgtgatatg |
|  | IVmiR*a | gaAATATAGTTACAGCTGCGTTCtctacatatatattcct |

**Supplementary Table S2**

| Primers used for qRT-PCR in this study (5 '-3 ') | | |
| --- | --- | --- |
| AT1G54080 | ACATTCTCTGAGGCAAGCAGGTCT | ATGGGCCGCAGCTACATTCTGATA |
| AT1G28660 | ATGTTCCGCAGCGTGTCTTACTCA | TCATTGAGCCGTGGGATACAACCT |
| AT5G19390 | GACAACGAATTGCAAAGGAGGCCA | CTTTCAGCTTGCAGCTGCTCTTGT |
| AT2G36720 | TCAACTAGTGCTCCTGCGTGCATA | AAGCCTTCCCAACCAAAGCTTGAC |
| AT1G07930 | TCAAGCCTGGTATGGTTGTGACCT | TGTCACCTGGAAGTGCCTCAAGAA |
| AT1G10090 | GCAAAGCTGGTTGCACTTCTCCTT | AAGCACTGGGACAGGATTTCACCT |
| At4g05050 | CCCTAACGGGAAAGACGATTAC | CGGAGGAATACCCTCCTTATCT |
| AT3G01600 | GTGTCGCTGGAGATGAAGAA | TTGGACCACATCTTCCCTAAAG |
| AT4G30680 | AGGAATGGTTGTCTGCTCTATG | AACCAAACTCCCAAGGATCTC |
| AT5G11170 | GACTTGGTAGGAAGAGGGATTG | TGCCAACCCTGTGAAGATAG |
| AT3G08580 | GCCTCTTCCCTTCTGTTTGT | CTTCCACCACCTCCTTTCTTT |
| At3g57480 | CCAACATCACCTGGGAGAAA | CTGCATCTAGGAACAGGACATT |
| AT5G18250 | GTTGATCCGTTCATCAGGGATAG | GTAACCACATGGGTCCTTTGA |
| AT5G54940 | CAGATGCACCAGGAGCTAAA | ATCCTCTCGTAGCTGTACTCTT |
| AT2G04230 | TAGACTTTCCTTCTCGGGTTTG | GACTAGGGCAGCCACATAAA |
| AT3G52590 | AGCAGAGATTGATCTTCGCCGGAA | TGGGTGAAGACGAGCATAGCACTT |
| AT4G32010 | GTCGTCCTTAACTTCTGCTAGG | GTGCCTCCTCCCAAGTTATT |
| AT5G58000 | GATGTGGAGGGATGTATCAAGG | GTACGCTACCACCGATATGAAG |
| AT2G27340 | CTTCCACTGCCTGCAATCT | GGTCCCTAATGCTTCCCATAC |
| AT2G22090 | CTCAGCAACAGCAGGTACAA | CCAACATCCCAGCTCCATAAA |
| AT2G26860 | GCGTGCGTGAGCTGATTA | GACGAGCGATGTGCAAGTAT |
| AT4G22820 | AGGCATTTGTGCTGAGGAA | GAAGAGACTACGAGGAGGAGAA |
| AT3G13062 | CCGCAACAGAGGAAGTATGT | CCTGGTGAACCATGTGTATCT |
| AT3G43600 | AGCGTCAACCACTGCAATA | GAGGCATGGAAACCGGATAA |
| AT4G37020 | CCATGGCTTCCGATTGTAGT | GTACAAAGCGGCTAAGGAAATG |
| AT1G21110 | GGATATTGGATCCCTTGCTTCT | CTCCTTCTAGCACCACATCTTT |
| AT1G03360 | GACGCAGAAGGTTAGGTTTGA | GTCATGGTTGACGGGAATAGAG |
| AT1G18710 | GAGAAAGGGAATCGACCCTATG | GGTGGAAGAGTTACCACAATCT |
| AT3G14440 | GCTGCGGTTTCTGGGAGAT | GTCGGAGCTTTGAGAAGACGAT |
| AT1G30100 | CCTCCGTTAGTTTCACCAACACT | GGTGTGTCGGAGACGGAGTT |
| AT3G24650 | ACGTCAGCAGGTGGTACCAG | GGCAAGTGTGTCTCAGCTTC |
| AT2G40220 | ATGGACCCTTTAGCTTCCCAAC | CTTTGCGTTTGCGTTGAGCG |
| AT1G78390 | GGAAAACGCCATGATCTCACA | AGGATCCGCCGTTTTAGGAT |
| ATCG00490 | ATTCGGTGGAGGAACTTTAGGCCA | TCACAAGCAGCAGCTAGTTCAGGA |
| ATCG00020 | ATGGCTATACAACGGCGGTCCTTA | GCAACAGCAATCCAAGGACGCATA |
| ATCG00120 | AAAGCTTCTTCCGTGGCTCAGGTA | TCAGCCAAGGCTGCTCCTGTATAA |
| ATMG00070 | ACCAGTGCAGACGAAGTAACACGA | ACGGCGTAGATCCGGATGATTGAT |
| AT5G46110 | TTGCTGTCTCCTTCACTCACACCA | TAGCACTGATGAAACCGAGCCAGT |
| AT5G48300 | TCAAATCCACCGTCTCCCGTCTTT | AGCTGGTTTCGCTCTCTTCTTCGT |
| AT5G19220 | TGGACTTGGCTTTGGAGATGGCTA | TCTGCTGTACCTTGGAACCACCTT |
| AT3G27690 | CGGAGAATACCCTGGAGACTA | CCCATCTACTGTGGATCACTTC |
| ATCG00340 | TGGCGTGGTTATTGGCAGGAATTG | AATTTGCCCGATGTGGAGGCAATC |

| AT3G15170 | TACAAAGGCAGAGCTCCTAAAG | AGCGGAGGAGGAAATGTAATG |
| --- | --- | --- |
| AT5G53950 | CGGGAGAATCATGGGAGATATG | GGTTGATCGGGTCGGTATAAA |
| AT1G76420 | CGAAGATGGGAGAGAGAGAGT | GCTTTCCAGTATCCAGCAGTAG |
| AT4G37800 (XTH7) | CGCAGATTGTCCCGCTAAT | GTTCACTCGGACCCATCTATAAC |
| AT4G30280 (XTH18) | GCAAAGCCGAGGTTCAAATG | CAATCTCATCCCACGTAGTTCC |
| AT4G30290 (XTH19) | TATGGGAAGCAGAGCATTGG | CACATCCCTCGACATTGTAGTT |
| AT1G20190 (EXPA11) | TGTAGGAGGAGCAGGTTCTATT | CCAGTTACGAGACATGGCTAAC |
| AT2G37640 (EXPA3) | GGAGTTAGCGTAAAGGGATCAA | GAGTGATTGGCCGATGAGAA |
| AT1G26770 (EXPA10) | GGTTGGTGTAATCCTCCTCTTG | GGAACCCTTCTGTAGGAAACAG |
| At4g34270 | GTGAAAACTGTTGGAGAGAAGCAA | TCAACTGGATACCCTTTCGCA |
| AT3G07750 (RRP42)  AT3G45970 (EXPL1)  AT3G24220(NCED6)  AT5G44120 (CRA1)  AT5G56870  AT5G54060  MRP  snoRNA31 | GGAGCAGGAGCTGGAATAAA  GCTATCCAGTTCAGGTTCGTAG  CGTTATTCCTATGGAGCAGAATCG  TAGCCGGAAACAACCCACAAG  TGGAGGTGCGGTACCATACA  ACGAGGCGATTGGCTCTTT  TCGGCGGAGAGACCACAT  GCAAAATTAATCTAATCCAACACTTGAT | CCCATCCGAACTAATGACAAGA  AACACCGGCGTCGTAAAT  GGAGCGAAGTTACCTGATAATTGAA  GAAGTTGCTGTGCTGTCTGAAG  GGAACCTCCTTTTTGGATAAATCG  CGATTGCGTCGCAGTTTCT  CTTGCGGTGGAGAGATTCAAA  TGGAGATGCACATAGTATCTCTCAGA |

**Supplementary Table S3**

The levels of transcripts up-regulated in reported mutants of *rrp41^iRNAi^*, *rrp4^iRNAi^* and *rrp41l* in WT, *a42-1* and *a42-3* plants.

qRT-PCR analysis of transcripts (shown in fold change) in 6-week-old WT, *a42-1* and *a42-3* rosette leaves 7 to 12 is shown. The expression level in the WT was set at 1. The means of three replicates of qRT-PCR and SD values are shown. Similar results were obtained when qRT-PCR was performed using a second set of samples. Dashes indicate transcripts that were not detected.

| AGI | Symbol | WT | *a42-1* | *a42-3* |
| --- | --- | --- | --- | --- |
| Transcripts up-regulated in *rrp41^iRNAi^* | | | | |
| AT5G56870 |  | 1 | 0.82±0.15 | 1.47±0.17 |
| AT1G54080 |  | 1 | 1.45±0.06 | 1.48±0.11 |
| AT1G28660 |  | 1 | 1.70±0.17 | 1.35±0.17 |
| AT5G19390 |  | 1 | 0.88±0.05 | 0.99±0.05 |
| AT2G36720 |  | 1 | 0.54±0.07 | 0.69±0.18 |
| AT5G01930 |  | 1 | 2.33±0.22 | 1.71±0.14 |
| AT1G10090 |  | 1 | 1.25±0.07 | 1.56±0.23 |
| AT4G05050 |  | 1 | 0.92±0.13 | 0.70±0.09 |
| AT3G01600 |  | 1 | 0.66±0.13 | 0.48±0.08 |
| AT4G30680 |  | 1 | 1.11±0.03 | 1.33±0.09 |
| AT5G11170 |  | 1 | 1.02±0.13 | 1.25±0.20 |
| AT3G08850 |  | 1 | 1.27±0.18 | 1.32±0.29 |
| AT3G57480 |  | 1 | 0.67±0.10 | 0.87±0.13 |
| AT5G18250 |  | 1 | 0.97±0.16 | 1.12±0.16 |
| AT5G54940 |  | 1 | 0.86±0.10 | 0.93±0.08 |
| AT2G04230 |  | 1 | 0.89±0.08 | 1.07±0.09 |
| Transcripts up-regulated in *rrp4^iRNAi^* | | | | |
| AT5G54060 |  | 1 | 0.71±0.19 | 0.97±0.1 |
| AT3G52590 |  | 1 | 1.04±0.07 | 0.75±0.06 |
| AT4G32010 |  | 1 | 0.97±0.35 | 1.12±0.43 |
| AT5G58000 |  | 1 | 1.26±0.38 | 0.91±0.20 |
| AT2G27340 |  | 1 | 0.87±0.14 | 0.76±0.09 |
| AT2G22090 |  | 1 | 1.14±0.20 | 0.86±0.11 |
| AT2G26860 |  | 1 | 1.14±0.17 | 0.78±0.15 |
| AT4G22820 |  | 1 | 1.00±0.29 | 0.80±0.16 |
| AT3G13062 |  | 1 | 0.87±0.16 | 0.86±0.11 |
| AT3G43600 |  | 1 | 0.99±0.27 | 0.97±0.37 |
| AT4G37020 |  | 1 | 1.28±0.14 | 1.08±0.04 |
| AT1G21110 |  | 1 | 0.42±0.04 | 0.67±0.07 |
| AT1G03360 |  | 1 | 2.51±0.04 | 1.33±0.10 |
| AT1G18710 |  | 1 | 1.40±0.55 | 3.74±0.54 |
| Transcripts up-regulated in *rrp41l* | | | | |
| AT3G14440 | NCED3 | 1 | 1.66±0.06 | 1.41±0.06 |
| AT1G30100 | NCED5 | 1 | 0.40±0.07 | 0.80±0.21 |
| AT5G44120 | CRA1 | 1 | 1.15±0.72 | 1.37±0.4 |
| AT3G24650 | ABI3 | - | - | - |
| AT2G40220 | ABI4 | - | - | - |
| AT3G24220 | NCED6 | - | - | - |
| AT1G78390 | NCED9 | - | - | - |

**Supplementary Table S4**

The levels of transcripts up-regulated in mutants of *a42-1* in WT, *rrp41l* seedlings.

qRT-PCR analysis of transcripts (shown in fold change) in 4-day-old WT, *rrp41l* seedlings is shown. The expression level in the WT was set at 1. The means of three replicates of qRT-PCR and SD values are shown. Similar results were obtained when qRT-PCR was performed using a second set of samples.

| AGI | Symbol | WT | *41l* |
| --- | --- | --- | --- |
| AT4G37800 | XTH7 | 1 | 0.72±0.04 |
| AT4G30280 | XTH18 | 1 | 1.49±0.14 |
| AT4G30290 | XTH19 | 1 | 1.41±0.06 |
| AT2G37640 | EXPA3 | 1 | 1.13±0.17 |
| AT1G26770 | EXPA10 | 1 | 0.9±0.11 |
| AT1G20190 | EXPA11 | 1 | 0.91±0.05 |
